# Residual paralysis caused by 50 mg rocuronium after reversal with 4 mg/kg sugammadex: a case report

**DOI:** 10.1186/s12871-021-01379-6

**Published:** 2021-05-20

**Authors:** Kohji Uzawa, Hiroyuki Seki, Tomoko Yorozu

**Affiliations:** Department of Anesthesiology, Kyorin University School of Medicine, 6-20-2, Shinkawa Mitaka, Tokyo, 181-8611 Japan

**Keywords:** Sugammadex, Rocuronium, Residual paralysis, Neuromuscular monitoring, Case report

## Abstract

**Background:**

Rocuronium-induced neuromuscular blockade can be quickly and completely reversed by administration of an optimal dose of sugammadex. Sugammadex antagonizes rocuronium-induced neuromuscular blockade by encapsulating rocuronium. Herein, we report a case of residual neuromuscular paralysis in which the recommended dose of sugammadex (4 mg·kg^− 1^) failed to antagonize a rocuronium-induced blockade.

**Case presentation:**

A 71-year-old man (body mass index: 26.7 kg·m^− 2^) underwent endoscopic submucosal dissection of early-stage gastric cancer. He had no known factors that may have affected the effects of rocuronium and sugammadex. He received rocuronium (50 mg; 0.7 mg·kg^− 1^) for anesthesia induction. No additional rocuronium was administered during the 71-min procedure. Ninety-four minutes after rocuronium administration, neuromuscular monitoring showed 20 twitches in response to post-tetanic count stimulation. The train-of-four (TOF) ratio was not measurable despite sugammadex (280 mg; 4 mg/kg) administration, although four weak twitches in response to TOF stimulation appeared in 3 min. The TOF ratio became detectable following administration of an additional dose of sugammadex (120 mg; 1.7 mg·kg^− 1^), and it recovered to 107% 8 min after the second dose. The patient opened his eyes; moved his neck, arms, and limbs; and regained consciousness. The trachea was extubated and the patient was transferred to the ward.

**Conclusions:**

Neuromuscular monitoring should be used if a neuromuscular blockage agent is administered, even if the recommended dose of sugammadex is administered.

## Background

Sugammadex rapidly and completely reverses neuromuscular blockade by encapsulating rocuronium. Although sugammadex can reverse any depth of rocuronium-induced blockade, its optimal dose should be determined based on objective monitoring of neuromuscular blockade depth. For antagonizing deep rocuronium-induced neuromuscular blockade (post-tetanic count [PTC] ≥ 2; train-of-four [TOF] count = 0), 4 mg/kg sugammadex administration is recommended by the manufacturer’s instructions, Miller’s anesthesia textbook, a recent review article, and online resources (Table 1) [1–4]. Anesthesiologists calculate the dose of sugammadex according to these recommendations, as administration of an inadequate amount of sugammadex may result in adverse respiratory events due to residual paralysis, while an excessive dose may increase the risk of hypersensitivity [5, 6]. Herein, we report a case of residual neuromuscular paralysis in which 4 mg/kg sugammadex was inadequate to antagonize rocuronium-induced blockade after spontaneous recovery to the appearance of 20 twitches in response to PTC stimulation. The patient provided written consent for the publication of this case report. This article adheres to the CARE (case report) guidelines.

**Table 1:** Recommended dose of sugammadex by reliable sources

Source	Description
Manufacturer’s instructions [1]	4 mg/kg is recommended if spontaneous recovery of the twitch response has reached 1–2 PTCs and there are no twitch responses to TOF stimulation. 2 mg/kg is recommended if spontaneous recovery has reached the reappearance of the second twitch in response to TOF stimulation.
Miller’s anesthesia textbook [2]	Three different doses of sugammadex are recommended according to the level of block. A large dose (16 mg/kg) is given during intense block (no response to PTC stimulation), a medium dose (4 mg/kg) during deep block (two or more responses to PTC), and a low dose (2 mg/kg) during moderate block (two or more responses to TOF stimulation).
Review article [3]	For reversal of moderate block (TOF count, 1–3), a dose of 2 mg/kg is recommended; for reversal of deep block (PTC ≥ 1), a dose of 4 mg/kg is recommended.
Online resource [4]	Deep block (at least 1–2 post-tetanic counts and prior to the second twitch following TOF stimulation): 4 mg/kg as a single dose. Moderate block (after appearance of the second twitch following TOF stimulation): 2 mg/kg as a single dose.

*PTC* Post-tetanic count, *TOF* Train-of-four

## Case presentation

A 71-year-old man (height, 163.4 cm; body weight, 71.2 kg; body mass index [BMI], 26.7 kg·m^− 2^) was scheduled for endoscopic submucosal dissection of early-stage gastric cancer. The patient had type 2 diabetes mellitus and had been on insulin therapy for 30 years. He had a history of total thyroidectomy 8 years ago and laparoscopic cholecystectomy 6 years ago. He was euthyroid (free T4, 1.24 ng·dl^− 1^; free T3, 2.06 pg·dl^− 1^; TSH, 0.586 μIU·ml^− 1^) with oral levothyroxine. Preoperative laboratory test results showed reduced estimated glomerular filtration rate (63.0 mL·min^− 1^), elevated hemoglobin A1c level (7.6%), and glucosuria (4+) due to sodium-glucose co-transporter-2 inhibitor use. Laboratory investigation results showed no other abnormalities.

In the operating room, routine monitoring including electrocardiography, noninvasive blood pressure measurement, and pulse oximetry was performed. After preoxygenation, general anesthesia was induced with 300 mg thiopental, 50 mg (0.7 mg·kg^− 1^) rocuronium, and 0.15 μg·kg^− 1^·min^− 1^ remifentanil. After tracheal intubation, the patient was mechanically ventilated to maintain an end-tidal carbon dioxide between 35 and 40 mmHg. Anesthesia was maintained with an end-tidal sevoflurane concentration of 1.2% and 0.1–0.15 μg·kg^− 1^·min^− 1^ remifentanil. Intraoperatively, the patient was administered 750 mL of crystalloids, and his urine output was 118 mL. The surgery was completed uneventfully and anesthesia was terminated in 71 min. No additional rocuronium was administered after anesthesia induction. Ninety-four minutes after rocuronium administration, neuromuscular monitoring (Intellivue NMT module®, Philips Japan, Tokyo) was applied and showed a TOF count of 0 and 20 twitches in response to PTC stimulation in the adductor pollicis muscle. The end-tidal sevoflurane concentration was 0.1%. Sugammadex (280 mg; 4 mg/kg) was administered, and four twitches in response to TOF stimulation appeared in 3 min. However, the twitches were still weak, and no spontaneous breathing or movement was observed. The TOF ratio was not measurable. Therefore, an additional dose of sugammadex (120 mg; 1.7 mg·kg^− 1^) was administered 5 min after the first dose. Five minutes after the second dose of sugammadex, the TOF ratio recovered to 92%, and 8 min after the second dose, it recovered to 107%. The patient opened his eyes; moved his neck, arms, and limbs; and regained consciousness. The trachea was extubated and the patient was transferred to the ward.

When we interviewed the patient at a postoperative follow-up, he told us he had felt “numbness” on awakening from anesthesia after the cholecystectomy he underwent 6 years ago and that the anesthesiologists “did something” to cure it. He and his family also had no history of neuromuscular disorders. He was discharged without complications on the fifth postoperative day.

## Discussion and conclusions

In this case, full recovery from neuromuscular blockade was not achieved with the recommended dose of sugammadex. It was unclear whether this was because the dose of sugammadex administered was inadequate or the recovery was delayed. Although rare, there have been reports of unexpectedly large amounts of sugammadex needed to reverse rocuronium-induced paralysis. Fernandes et al. reported a case of a patient with obesity (BMI: 37.2 kg·m^− 2^) and myasthenia gravis in whom a total of 800 mg (7.27 mg·kg^− 1^) of sugammadex and 2 mg of neostigmine was required to antagonize 25 mg of rocuronium [7]. Kiss et al. reported another case of a patient with obesity (BMI: 32.0 kg·m^− 2^) and myasthenia gravis in whom a total of 17.34 mg/kg of sugammadex did not achieve complete recovery from 50 mg rocuronium-induced paralysis [8]. Ortiz-Gomez et al. reported a case where sugammadex failed to reverse rocuronium-induced neuromuscular blockade [9]. In that report, the patient was highly obese (BMI: 37.6 kg·m^− 2^) and hypertensive, but otherwise healthy. After tracheal intubation with 100 mg of succinylcholine, 70 mg of rocuronium (1.0 mg·kg^− 1^ for ideal body weight) was administered followed by continuous infusion during a six-hour surgery, resulting in a total dose of 153 mg of rocuronium. To antagonize rocuronium, a total of 1120 mg (9.74 mg·kg^− 1^) of sugammadex was administered, but complete recovery was not achieved. Because sugammadex and rocuronium bind in a 1:1 M ratio, 3.57 mg of sugammadex (molecular weight: 2178 kDa) is needed to encapsulate 1.0 mg rocuronium (molecular weight: 610 kDa) [10]. In these reports, although a sufficient amount of sugammadex was administered, the patients were highly obese, had neuromuscular disorders, or had received long-term rocuronium administration, which might have affected the effects of neuromuscular blocking agents. Our patient had neither neuromuscular disorders nor other factors that may affect the effect of rocuronium or sugammadex, such as hypoalbuminemia, liver dysfunction, and dehydration. Although the estimated glomerular filtration rate was slightly low, the serum creatinine level was normal and no albuminuria was noted. For anesthesia induction, 50 mg of rocuronium was administered, following which no additional dose was administered. Thus, 178.5 mg of sugammadex, which is much less than what we administered, was theoretically needed to encapsulate 50 mg of rocuronium. In addition, the dose of sugammadex administered was calculated based on the patient’s actual body weight. It has been shown that ideal body weight-based doses can result in inadequate reversal or recurarizaion, especially in patients with obesity [11]. Therefore, it is unlikely that the amount of sugammadex administered to our patient was insufficient.

On the other hand, it may be possible that the recovery after sugammadex administration was delayed. In most cases, sugammadex antagonizes rocuronium within a couple of minutes. However, there have been reports of the outliers. A post hoc analysis of data from a multi-center trial showed that reversal of the TOF ratio to 0.9 occurred within 5 min of 4 mg/kg sugammadex administration in more than 80% of patients regardless of neuromuscular blockade depth [12]. However, there was a considerably wide inter-individual variation; the time to achieve a TOF ratio of 0.9 varied from 0.8 to 22.3 min when patients with no twitch in response to TOF stimulation received 4 mg/kg sugammadex [12]. Another study showed a wide variation in the speed of reversal following sugammadex administration [13]. In that study, patients received 0.6 mg/kg of rocuronium followed 3 min later by 4 mg/kg of sugammadex. In most patients, the TOF ratio recovered to 0.9 within 5 min. However, one patient had a recovery time of 24.6 min [13]. Furthermore, substantial individual variation is known to exist in the duration of action of rocuronium. In one study, the median time for reappearance of T1 after 0.9 mg/kg rocuronium administration was 33.8 min [14]. It ranged from 16.2 to 52.9 min, indicating a wide variation in the duration of action. However, even after 0.9 mg/kg rocuronium administration, all patients recovered to a TOF count of 1 in 60 min. In our patient, the TOF count was not detected at 94 min after 0.7 mg/kg rocuronium administration. It is well recognized that neuromuscular monitoring in diabetic patients with peripheral polyneuropathy can be inaccurate [15]. Furthermore, it has been shown that spontaneous recovery from rocuronium-induced neuromuscular block is prolonged in diabetic patients, even in the absence of complications [16]. Although the patient was not diagnosed with diabetic neuropathy, his history suggests prolonged neuromuscular blocking agent action when he previously underwent anesthesia. Diabetes or patient variability in response to muscle relaxants and reversal agents could cause residual paralysis in this case. Although it is unclear if the speed of reversal following sugammadex administration was slow in this case, waiting longer might have been an option, as an excessive dose of sugammadex may affect the effectiveness of rocuronium in case of reoperation or reintubation.

In conclusion, neuromuscular monitoring should be used if a neuromuscular blockage agent is administered, even if the recommended dose of sugammadex is administered.

## Data Availability

Not applicable.

## Abbreviations

BMI: Body mass index
PTC: Post-tetanic count
TOF: Train-of-four
